# Ovarian tissue cryopreservation in the pediatric with rare diseases- experience from China’s first and the largest ovarian tissue cryobank

**DOI:** 10.3389/fendo.2023.1137940

**Published:** 2023-04-03

**Authors:** Xiangyan Ruan, Jiaojiao Cheng, Juan Du, Fengyu Jin, Muqing Gu, Rui Ju, Yurui Wu, Long Li, Yuejiao Wang, Lingling Jiang, Yu Yang, Yanqiu Li, Zecheng Wang, Jun Ma, Mingzhen Zhang, Alfred O. Mueck

**Affiliations:** ^1^ Department of Gynecological Endocrinology, Beijing Obstetrics and Gynecology Hospital, Capital Medical University, Beijing Maternal and Child Health Care Hospital, Beijing, China; ^2^ Department for Women's Health, University Women’s Hospital and Research Center for Women’s Health, University of Tuebingen, Tuebingen, Germany; ^3^ Department of Thoracic Surgery and Surgical Oncology, Children’s Hospital, Capital Institute of Pediatrics, Beijing, China; ^4^ Department of Pediatric Surgery, Children’s Hospital, Capital Institute of Pediatrics, Beijing, China

**Keywords:** ovarian tissue cryopreservation, children, fertility preservation, rare diseases, hematopoietic stem cell therapy

## Abstract

**Background:**

There is limited information about the efficacy of ovarian tissue cryopreservation (OTC) in children. In the present study, we report eight patients with rare diseases who underwent OTC in China’s first and largest ovarian tissue cryobank.

**Procedure:**

Data from girls with rare diseases who underwent OTC between September 2020 and November 2022 were retrospectively analyzed. We also compared the number of cryopreserved cortex pieces, follicle number, and AMH in those with rare diseases and age-matched children with non-rare diseases who also underwent OTC in our cryobank.

**Results:**

The median age of the children was 5.88 ± 3.52 (range 2-13) years old. Unilateral oophorectomy was undertaken *via* laparoscopy in all of the children. The diseases in the 8 patients were: 4 mucopolysaccharidoses (MPS I two cases, IVA two cases), 1 Diamond-Blackfan anemia (DBA), 1 Fanconi anemia (FA), 1 hyperimmunoglobulin E syndrome (HIES), 1 Niemann-Pick disease. The number of cryopreserved cortex pieces was 17.13 ± 6.36, and the follicle count per 2 mm biopsy was 447.38 ± 524.35. No significant difference in age, the count of cryopreserved cortex pieces, follicle number per 2 mm biopsy, and AMH level was seen between the 20 children with non-rare diseases and those with rare diseases.

**Conclusions:**

The reports help practitioners counsel girls with rare diseases about fertility preservation. The demand for OTC in pediatrics will likely grow as a standard of care.

## 1 Introduction

Diseases with an incidence of fewer than 1/10,000 newborns, a prevalence of less than 1/10,000, and a number of patients less than 140,000 are classified as rare diseases (RD). As of February 2022, there are more than 7,000 known rare diseases worldwide, accounting for about 10% of all human diseases. About 72%-80% of rare diseases are caused by structural changes or abnormal regulation of genetic material (1). China has over 20 million rare disease patients, with over 200,000 new patients yearly (2). Around the world, the treatment of rare diseases is probably the most significant medical challenge facing humanity today. Treatment of some rare diseases requires hematopoietic stem cell therapy (HSCT) (3). Advances in HSCT technology and supportive care have increased the number of long-term survivors (4), so fertility preservation (FP) for these patients is also essential.

Ovarian tissue cryopreservation (OTC) is the only FP method for prepubertal girls (5). OTC includes laparoscopic surgery to remove part or the whole ovary, followed by transport to the ovarian tissue cryobank for cryopreservation and storage (6, 7). Professional institutions must have the necessary equipment, trained personnel, and sufficient frozen stock to conduct OTC safely and with consistent quality. More than 200 babies worldwide have been born through this technique, including reports of frozen ovarian tissue collected before puberty and frozen-thawed ovarian tissue transplantation (OTT) after puberty (8–10). In 2019, the American Society for Reproductive Medicine (ASRM) recommended that OTC technology no longer be considered experimental (11). Minimal complications have been reported after laparoscopic surgery (12).

The Oncofertility Consortium’s National Physicians’ Cooperative (ON-NPC) published its experience with OTC in 114 girls<15 years old in 2018 (13). Germany’s UniCareD cryobank stored frozen ovarian tissue from 104 girls with a mean age of 14 years between 2018 and May 2022 (14). Our center has cryopreserved ovarian tissue from more than 50 children (12).

Studies on FP in RD are limited. Because of the high incidence of POI for patients with genetic abnormalities, such as galactosemia and Turner syndrome (TS), experts recommend starting FP as soon as possible (15–19). FP counseling is also needed for children with RD who plan to undergo HSCT. This paper reports on the cryopreservation of ovarian tissue in 8 patients with RD to give medical workers more information and confidence and introduce patients to the FP center for consultation. A multidisciplinary team should always be involved in treating and managing RD patients.

## 2 Methods

### 2.1 Ethics statement

The Ethics Committee of Beijing Obstetrics and Gynecology Hospital, Capital Medical University, approved the provision of centralized OTC (2017-KY-020-01; March 15, 2017). Ovarian tissue was received from different hospitals and transported to the centralized cryobank. The parents of each patient signed an agreement and informed consent for their child.

### 2.2 Ovarian tissue retrieval, transportation, and preparation

Eight girls with RD underwent OTC in our cryobank between September 2020 and November 2022 (mean ± SD, range, 5.88 ± 3.52 years, 2-13 years). Unilateral oophorectomy was undertaken *via* laparoscopy in the eight children. No complications were reported after laparoscopic surgery in the eight children.

After retrieval, the ovarian tissue was immediately put into the cooled Custodiol (HTK). The temperature was maintained at 4-8 °C during ovarian tissue transportation. The mean temperature on reaching the cryobank was 5.44 °C, and the transport time was no more than 12 hours. In a sterile laminar flow cabinet, ovarian tissue was prepared at HTK solution, maintained at 4 °C. The ovarian cortex was handled to be 1 mm thick, cut to 6 mm x 3 mm slices, and cryopreserved. After slow programmed freezing, the tubes were stored in a liquid nitrogen tank with a gas phase. In ovarian tissue preparation, standardized cortical biopsies with a diameter of 2 mm from different areas were evaluated for follicle density and viability assay. The procedures were according to the previous publications (12, 20).

Twenty age-matched children who underwent OTC because of non-rare diseases were selected to compare the number of cryopreserved ovarian cortex pieces, follicle number per 2 mm biopsy, and the level of AMH between patients with RD and those with non-rare diseases. They did not undergo gonadotoxic treatment before OTC.

### 2.3 Hormone levels before OTC

The levels of follicle-stimulating hormone (FSH), luteinizing hormone (LH), and AMH in serum before OTC were evaluated. The details can be seen in our previous publication (15).

### 2.4 Analysis of follicle density

The number of surviving resting follicles was evaluated in biopsies by Calcein-AM assay, which method has been described in the previous publication (21).

### 2.5 Statistical analyses

SPSS 22.0 was applied for analysis. The data following normal distribution were expressed by “mean ± Standard deviation (SD)”; otherwise, “mean ± Standard Error of Mean (SEM)”. The differences between groups were compared by independent sample t-test. *P* < 0.05 indicates that the difference was statistically significant.

## 3 Results

### 3.1 Characteristics

#### 3.1.1 Ages and diseases diagnosis

Characteristics of the 8 girls with OTC are shown in **Table 1**. The age of the 8 children was 5.88 ± 3.52 years old, range 2 to 13 years. The diseases in the 8 patients were: 4 mucopolysaccharidoses (MPS I two cases, IVA two cases), 1 Diamond-Blackfan Aanemia (DBA), 1 Fanconi anemia (FA), 1 hyperimmunoglobulin E syndrome (HIES), 1 Niemann-Pick disease. Ovarian tissue was cryopreserved because of planned HSCT.

**Table 1:** Patient Characteristics before OTC.

Patients	Disease	Age at OTC	Transport temperature	Number of cryopreserved cortex pieces	Follicle number per 2mm biopsy	FSH (IU/L) before OTC	LH (IU/L) before OTC	AMH (ng/ml) before OTC
Case 1	Diamond-Blackfan anemia	7	4.7	10	128	3.47	0.01	0.48
Case 2	Fanconi anemia	7	5.8	17	122	2.2	0	1.15
Case 3	Mucopolysaccharidosis I	5	7.1	21	229	–	–	2.65
Case 4	Mucopolysaccharidosis IVA	3	6.2	17	1581	4.31	0.02	1.31
Case 5	Mucopolysaccharidosis IVA	3	4.9	13	437	6.83	0	0.67
Case 6	Mucopolysaccharidosis I	7	4.0	20	851	1.22	0	4
Case 7	hyper Ig E syndrome	13	5.3	29	133	1.02	3.75	1.88
Case 8	Niemann-Pick disease	2	5.5	10	98	5.16	0.06	0.65

OTC, ovarian tissue cryopreservation; FSH, follicle-stimulating hormone; LH, luteinizing hormone; AMH, anti-Müllerian hormone.

#### 3.1.2 Ovarian tissue retrieval, transportation, cryopreservation, follicle number, FSH, LH, and AMH

**Figure 1:**
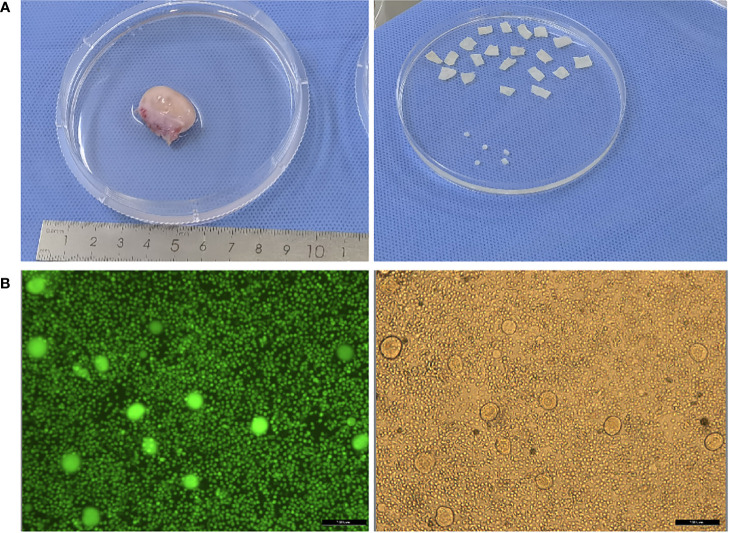
**(A)** Photos of ovary before and after ovarian tissue cortex preparation; **(B)** Detection of follicular activity in ovarian cortex.

Unilateral oophorectomy was undertaken *via* laparoscopy in the children. The temperature during transport to a centralized cryobank was 5.44 ± 0.96°C. The number of cryopreserved cortex pieces was 17.13 ± 6.36 (mean ± SD), the follicle number per 2 mm biopsy was 447.38 ± 524.35 (mean ± SD) (**Figure 1**). The FSH was 3.46 ± 2.14 IU/L (mean ± SD), LH was 0.55 ± 0.53 IU/L (mean ± SEM), and AMH was 1.60 ± 1.21 ng/ml (mean ± SD).

### 3.2 The comparisons between rare diseases and non-rare diseases

**Figure 2:**
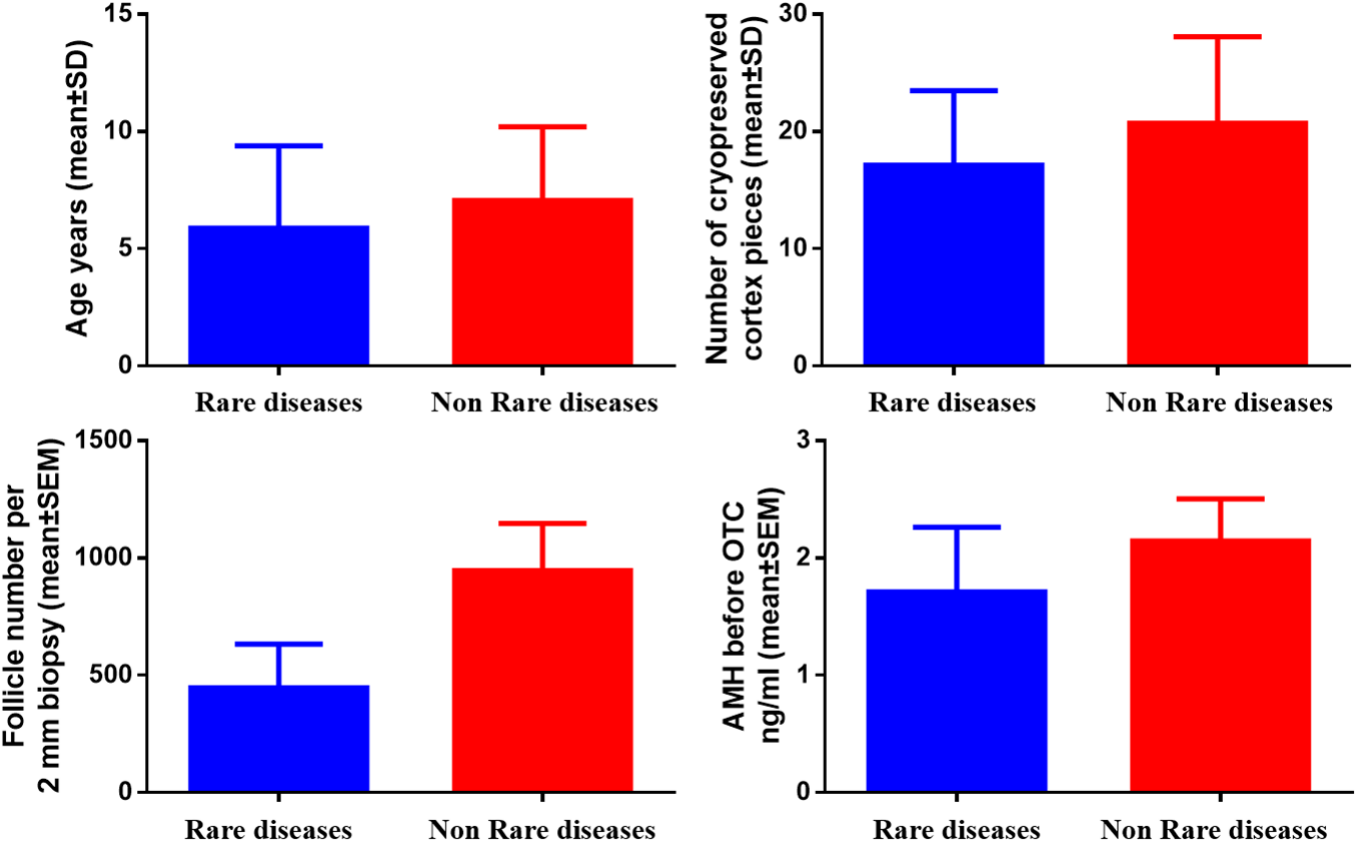
Comparison of age, number of cryopreserved cortex pieces, follicle number per 2 mm biopsy, and AMH in children with and without rare diseases.

In **Figure 2**, no significant difference in age, the number of cryopreserved cortex pieces, follicle number per 2 mm biopsy, and AMH level between 20 children with non-rare diseases and those with rare diseases (mean ± SD, 7.05 ± 3.16 vs. 5.88 ± 3.52, *P*=0.396; mean ± SD, 20.70 ± 7.39 vs. 17.13 ± 6.36, *P*=0.241; mean ± SEM, 947.35 ± 200.30 vs. 447.38 ± 185.39, *P*=0.153; mean ± SEM, 2.15 ± 0.36 vs. 1.60 ± 0.43, *P*=0.616, respectively).

## 4 Discussion

No studies have described OTC for DBA, FA, MPS-I, MPS-IVA, HIES, and Niemann-Pick disease. Patients with these diseases required high-dose chemotherapy with alkylating agents and/or total body irradiation as pre-treatment for HSCT. Therefore, the present study includes important information for OTC in children with RD.

With the development of Assisted Reproductive Technology (ART), embryos that do not carry explicit disease-causing genes can be transferred back to the maternal uterine cavity after embryo preimplantation genetic testing (PGT) for families with evident genes for RD/genetic disorders. This is an essential technical tool for the primary prevention and control of congenital disabilities and can stop the transmission of RD/genetic disorders in the family from the source and produce healthy offspring (22). Secondary prevention is prenatal screening, which requires amniocentesis during pregnancy to ensure again that the fetus does not carry the disease-causing genes. Tertiary prevention is post-birth screening.

DBA is a rare congenital intrinsic erythroid hypoplasia, with 7 cases/million live births, acknowledged in 2005 as the first human ribosomopathy (23, 24). The median age of diagnosis is two to three months, with 95% of the DBA cases diagnosed before two years and 99% before five years of age (25, 26). HSCT is safe and efficient in DBA and should be considered if a matched sibling or unrelated donor is available (26, 27). FA is a challenging disease, and HSCT is the only curative therapy for the hematologic complications associated with this disease (28). The favorable mean overall survival was 80.9%, and event-free survival was 79.3% (29).

MPS is a group of rare genetic diseases with abnormal glycosaminoglycan (GAG) catabolism (30). The overall incidence of MPS is estimated to be 1/100,000 live births, which varies according to region and race (31). HSCT has been used in patients with MPS I, II, IVA, VI, and VII, showing increased acceptance and therapeutic benefits (32). In 2011, a retrospective study evaluated the outcome of HSCT in 45 patients with MPS VI, with a 1-and 3-year survival rate of 66%. Patients who received HSCT had a longer life expectancy than those who did not receive treatment or enzyme replacement therapy (ERT) (33). The overall survival rate after transplantation was 90% (30). Standardized follow-up and a multidisciplinary team help accurately assess long-term post-transplantation outcomes and improve the quality of life (34).

HIES is primary immunodeficiency that results from heterozygous mutations in the signal transducer and activator of the transcription 3 genes. Some patients with HIES have been reported to be treated with HSCT. However, the efficacy of HSCT for autosomal dominant HIES is inconsistent (35). HSCT potentially benefits the severe phenotype of Niemann-Pick disease patients (36). However, pre-treatment with radiotherapy and high-dose chemotherapy before HSCT can seriously harm the ovaries, and 70~100% of young females develop premature ovarian insufficiency (POI) (37, 38).

Our study found no significant difference in ovarian size, follicle number, and AMH level before OTC between children with rare diseases and age-matched children with non-rare diseases. However, it did not include children with Turner syndrome who have accelerated follicle depletion prior to puberty (39–41). Also, further evaluation is necessary after increasing the sample size. The measurement of AMH levels is controversial because the assessment of ovarian reserve in children and adolescents using AMH levels has limitations (9, 42).

Ovarian size varies with age and pubertal development. The prepubertal ovary is smaller than the reproductive ovary; therefore, the entire unilateral ovary is usually removed (43). Many pediatric surgeons and gynecologists perform unilateral laparoscopic oophorectomy, using an ultrasonic advanced energy device to segment the ovarian artery and mesovarium. No major surgical complications have been observed with this technique (44).

With increasing evidence of live births and recovery of endocrine function, a British Fertility Society guideline concluded that prepubertal girls should be considered for OTC (45, 46). OTC and transplantation are now the only FP option for prepubertal girls (47, 48).

Our center is the first and largest ovarian tissue cryobank in China. Nearly 500 cases of ovarian tissue have been successfully preserved, 10 cases have been successfully transplanted, and the ovarian function has recovered after OTC and OTT (49). One patient with MDS successfully conceived naturally and delivered a healthy baby girl after OTC and OTT (50). With the cooperation of pediatrics, OTC in children in our center has increased in the last two years (12).

The limitation of this study is the lack of data regarding outcomes such as later fertility following the use of cryopreserved ovarian tissues. This should be mentioned when counseling by practitioners. The outcome in these patients will hopefully be reported on long-term follow-up. It should stimulate further research within this challenging scientific field.

## 5 Conclusion

To conclude, reporting information helps practitioners counsel girls with RD about FP and the preservation of ovarian endocrine function supported by OTC. The demand for OTC in pediatrics will likely grow as a standard of care.

## Data availability statement

The original contributions presented in the study are included in the article/supplementary material. Further inquiries can be directed to the corresponding author.

## Ethics statement

The studies involving human participants were reviewed and approved by Beijing Obstetrics and Gynecology Hospital, Capital Medical University. Written informed consent to participate in this study was provided by the participants’ legal guardian/next of kin. Written informed consent was obtained from the individual(s) for the publication of any potentially identifiable images or data included in this article.

## Author contributions

All authors qualify for authorship by contributing substantially to this article. XR: project leader, and project supervisor, evaluated the ovarian reserve function of each child, supervised and guided ovarian tissue biopsy, transport, preparation, cryopreservation, follow-up, interpretation of results, and provided critical comments and revised the first draft. JC: article preparation, ovarian tissue transportation, preparation, and cryopreservation. JD, FJ, and MG: ovarian tissue preparation and cryopreservation. RJ, YRW, and LL: biopsied ovarian tissue. YJW, LJ, YY, YL, ZW, JM, and MZ: ovarian tissue transportation, AM: experimental supervision, interpretation of results, and article revision. All authors reviewed the article’s final version and approved it for publication.
